# Solid diet manipulates rumen epithelial microbiota and its interactions with host transcriptomic in young ruminants

**DOI:** 10.1111/1462-2920.15757

**Published:** 2021-09-20

**Authors:** Jianmin Chai, Xiaokang Lv, Qiyu Diao, Hunter Usdrowski, Yimin Zhuang, Wenqin Huang, Kai Cui, Naifeng Zhang

**Affiliations:** ^1^ Feed Research Institute of Chinese Academy of Agricultural Sciences, Key Laboratory of Feed Biotechnology of the Ministry of Agriculture and Rural Affairs Beijing 100081 China; ^2^ Department of Animal Science, Division of Agriculture University of Arkansas Fayetteville AR 72701 USA

## Abstract

Solid diet supplementation in the early life stages of ruminants could improve rumen microbiota and tissue development. However, most studies focus on bacteria in the rumen content community. The microbiota attached on rumen epithelium are rarely investigated, and their correlations with rumen content bacteria and host transcripts are unknown. In this study, rumen digesta attached in the epithelium from goats in three diet regimes (milk replacer only, milk replacer supplemented concentrate and milk replacer supplemented concentrate plus alfalfa pellets) were collected for measurement of the epithelial microbiota using next generation sequencing. Correspondingly, the rumen tissues of the same animals were measured with the host transcriptome. The distinct microbial structures and compositions between rumen content and epithelial communities were associated with solid diet supplementation. Regarding rumen development in pre‐weaning ruminants, a solid diet, especially its accompanying neutral detergent fibre nutrients, was the most significant driver that influenced the rumen microbiota and epithelium gene expression. Compared with content bacteria, rumen epithelial microbiota had a stronger association with the host transcriptome. The host transcriptome correlated with host phenotypes were associated with rumen epithelial microbiota and solid diet. This study reveals that the epithelial microbiota is crucial for proper rumen development, and solid diet could improve rumen development through both the rumen content and epithelial microbiota.

## Introduction

Ruminants provide an abundant source of the animal protein to meet the growing needs of human nutrition worldwide (Malmuthuge *et al*., 2019). The rumen, a critical organ for ruminant production, converts low‐quality forage to volatile fatty acids (VFAs) and microbial protein for body requirement and high‐quality protein for human consumption by fermentation of symbiotic microbiota. Manipulation of the rumen microbiota is an effective approach to improve rumen fermentation and development (Eisler *et al*., 2014). It was reported that diet has profound impacts on the microbiota in rumen content, VFA production and early rumen development (Wang *et al*., 2016; Lin *et al*., 2019; Lv *et al*., 2019; Malmuthuge *et al*., 2019). Moreover, the ability to digest a high‐fibre diet is evidence of the functional rumen in young ruminants after birth, and early sold diet intervention can accelerate the process of rumen maturation. Therefore, understanding the use of a solid diet to improve rumen maturation is a beneficial feeding strategy for animal production. However, the interaction between diet effects, the rumen microbiome and epithelium development are still unclear, creating an information gap for understanding rumen developments from early solid diet supplementation.

Until now, most of the studies related to the rumen focused on the content microbiome, but limited research has investigated the microbiota attached on the rumen epithelium that are associated with rumen content microbiota and attached to epithelial walls. The rumen epithelium‐associated microbiota may play a critical role in rumen development and nutrient absorption because it is associated with rumen content microbiota and interacts directly with the host (Petri *et al*., 2018; Seddik *et al*., 2018; Malmuthuge *et al*., 2019). In a previous study, differing community structure between the rumen content and the epithelial microbiome was confirmed in cattle (Scharen *et al*., 2017), and the epithelium attached microbiota was affected by dietary carbohydrate composition (Petri *et al*., 2018). The epithelial microbiota playing important roles in maintaining host metabolic and gut development was also reported (Jiao *et al*., 2019). As we know, VFAs can diffuse directly across the ruminal epithelium, and ammonia can be absorbed through the ruminal wall (McCann *et al*., 2014). Therefore, the epithelial microbiota may be involved in the diffusion process of these nutrients, necessitating further research. Moreover, solid diet effects on the interactions of rumen content, the epithelial microbiome and host tissue mRNA are unknown, especially considering the critical roles of microbiota‐related communities on epithelium, developing a gap for understanding host microbiota interactions.

Therefore, to investigate the effects of solid diet on rumen microbiota and development, this pilot study fed goat kids with three distinct diet regimes: milk replacer only (MRO), milk replacer supplemented with concentrate solid diet (MRC), and milk replacer supplemented with (concentrate + alfalfa) solid diet (MCA). Goat kids were slaughtered and subsequently sampled on 60 days of age, and then we investigated the 16S rRNA gene sequences of rumen microbiota (both content and epithelium) and host transcriptome to explore the diet‐microbiota–host interactions.

## Results

**Fig 1:**
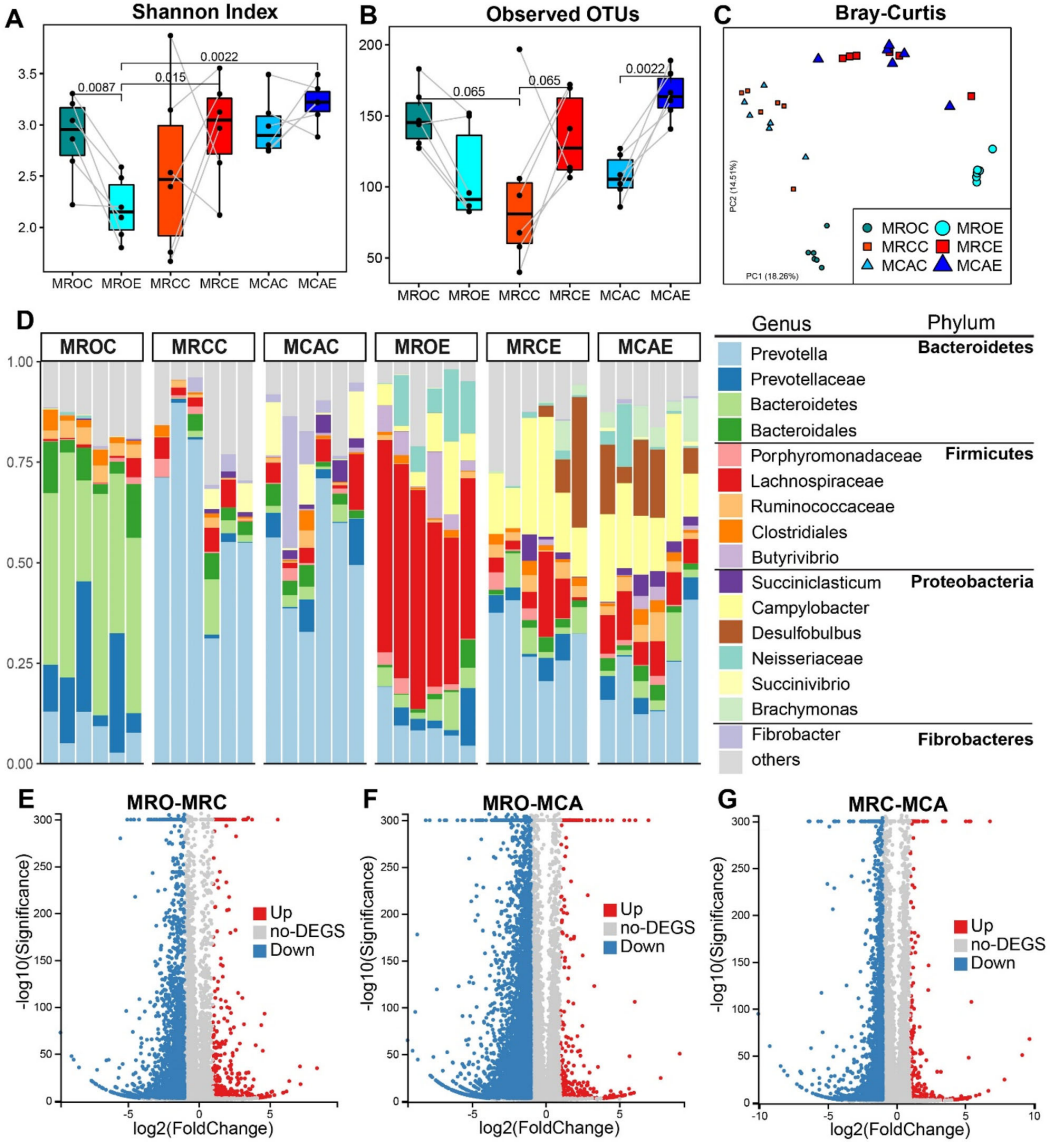
Solid diet intervention influences the rumen microbiota and epithelial transcriptome. A,B. Alpha diversity (Shannon index and the number of observed OTUs) of rumen microbiota. Content and epithelium samples from the same animals were connected using grey lines. *P* values of the Wilcoxon test were labelled over bars. C. Beta diversity of the rumen content and epithelial microbiota based on Bray–Curtis. One point represents one sample. D. Rumen microbial composition at the genus level. Each column represents a sample and each bar represents one bacterium. E‐G. The volcano plot of differently expressed genes in pair‐wise comparisons. MROC, MRCC and MCAC represent content samples in animals that received MRO, MRC and MCA diets, while MROE, MRCE and MCAE represent the epithelial microbiota from the three diets, respectively. The MRO treatment was fed only milk replacer, the MRC treatment was fed milk replacer with concentrate and the MCA treatment was fed milk replacer with concentrate plus alfalfa.

In this study, developing papillae were observed with increasing solid diet intake, especially alfalfa supplementation (Supporting Information Table S2, Fig. S1 A‐C). The length and width of papillae in goats with supplemented MRC and MCA diets were greater than in the MRO treatment, respectively (*P* < 0.05) (Supporting Information Table S2). Increased rumen weights in solid diet treatments (both MRC and MCA) were observed compared to MRO treatment goats (*P* < 0.001). Next, microbial diversity distinctions between rumen content and epithelial communities from the same subjects were determined. Rumen content microbial data were obtained from our previous study (Lv *et al*., 2019). Significant diversity and structure between content and epithelium microbial community were observed. In the MRO treatment, epithelium microbiota developed lower diversity (Shannon Index) compared with content microbiota (*P* = 0.0087). However, in the solid diet treatments (either MRC or MCA), epithelium richness was improved compared to content (*P* = 0.065, *P* = 0.0022). Considering beta diversity (Fig. 1C), distinct clustering of content and epithelium were observed in both goat kids supplied with and without solid diet [analysis of similarity (ANOSIM): MROC vs. MROE: *R* = 0.99, *P* = 0.001; MRCC vs. MRCE: *R* = 0.51, *P* = 0.001; MCAC vs. MCAE: *R* = 0.73, *P* = 0.003] (Supporting Information Table S3). Moreover, the microbiota in both rumen content and epithelial community was affected by a solid diet regime in the rumen content community. The number of observed OTUs in solid diet treatments decreased compared to the non‐solid diet group, while the Shannon index for the epithelium microbial community was greater for solid diet supplementation (Fig. 1A and B). Solid diet significantly influenced the beta diversity of microbial communities in both content and epithelium communities. For example, MRCE and MCAE contained a significant cluster when compared with MROE using Bray–Curtis distance (ANOSIM: *R* = 0.86, 0.92, *P* = 0.003, *P* < 0.001) (Fig. 1C). Overall, improved rumen development by solid diet were found, and significant differences between both rumen content and epithelial microbial communities were observed.

The significant changes of microbial composition in both content and epithelial communities affected by solid diet were observed. Genus *Prevotella* (Bacteroidetes phylum) was dominant across diet regimes and sampling communities although its relative abundances were different (Fig. 1D). In the MRO diet regime, unclassified *Lachnospiraceae* (Firmicutes phylum), *Campylobacter* and unclassified *Neisseriaceae* (Proteobacteria phylum) was abundant in the epithelium community when compared with its content community with *Bacteroidetes* genus. In the solid diet regime, *Prevotella* in epithelium was less abundant compared with content community, while genera including *Campylobacter*, *Desulfobulbus*, and unclassified *Lachnospiraceae* increased in their epithelium community. In addition, *Brachymonas* was specifically increased in epithelial community of the MCA diet. The microbial composition in rumen content and epithelial communities was different and affected by solid diet supplementation.

Transcriptomics of rumen tissue has been widely studied in ruminants to understand dynamics occurring in developing rumens associated with diet (Li *et al*., 2019; Lin *et al*., 2019; Yang *et al*., 2019). A total of 109 Gb of clean data were generated, with an average of 6.44 Gb per subject. Significantly different expressed genes (DEGs) were observed among diet regimes. Compared with the MRO diet, MRC and MCA treatments had 3062 and 6377 DEGs, respectively, which contained 432 and 451 up‐regulated genes, and 2630 and 5962 down‐regulated genes (Fig. 1E–G). Moreover, 425 shared DEGs can be found among the three pair‐wise comparisons, and 2065 common DEGs can be found between the comparisons of MRO‐MRC and MRO‐MCA treatments (Supporting Information Fig. S2), which indicated that a solid diet drove identical gene expression. These DEGs were associated with ‘system development’, ‘regulation of multicellular organismal processes’, ‘anatomical structure development’, ‘developmental processes’ and the ‘regulation of cell differentiation’ (Supporting Information Fig. S3A and B) based on gene ontology (GO) analysis. Additionally, 2908 DEGs (333 up‐regulated and 2575 down‐regulated genes) were found between MRC and MCA treatments (Supporting Information Fig. S1; Fig. 1D and E). These genes were related to the ‘microtubule cytoskeleton’, ‘organic cyclic compound binding’, ‘carbohydrate derivative binding’, and the ‘mitotic cell cycle’ (Supporting Information Fig. S3C). The present study found changes in the host transcriptome and the molecular mechanism influenced by solid diet supplementation.

**Fig 2:**
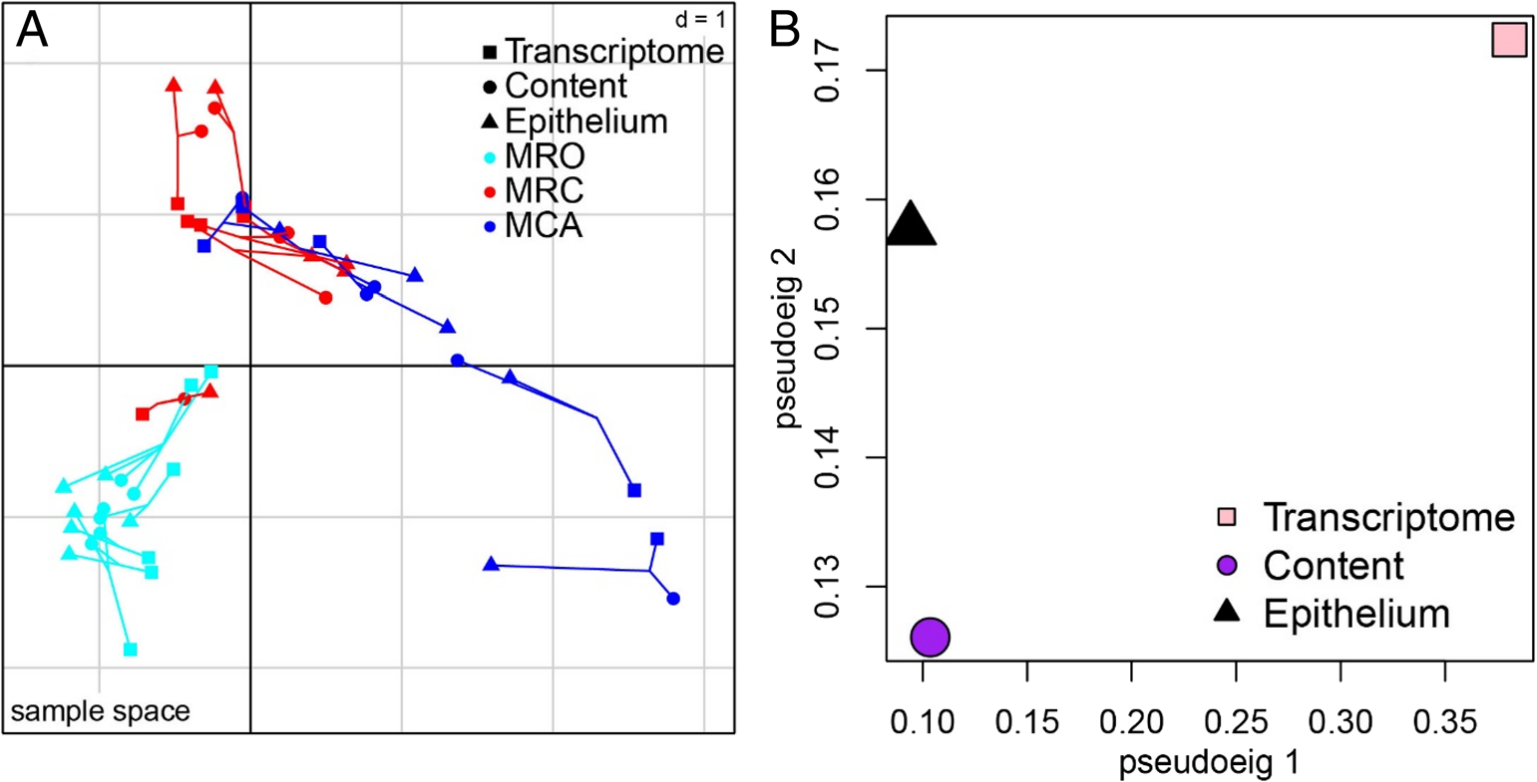
MCIA of rumen epithelium transcriptome, content and epithelial microbiota in goat kids. A: Sample space. The first two axes of the MCIA represent three communities including the rumen epithelium transcriptome, content and epithelial microbiota. Shapes represent samples in different communities. Samples from the same animal are linked by lines where the length of the lines is proportional to the divergence between the communities. Lines are added by a common point, representing the reference structure which maximizes covariance derived from the MCIA synthetic analysis. Colours represent the datasets from different diet regimes. The MRO group was projected on the negative sides of the first axis (PC1), while the communities of the MCA diet were projected on the positive side of PC1. B. Summarizing the concordance between communities by representing the pseudo‐eigenvalue space. The pseudo‐eigenvalue space indicates overall co‐structure between three communities across all diet regimes and shows which community contributes more to the total variance. The MRO treatment was fed only milk replacer, the MRC treatment was fed milk replacer with concentrate and the MCA treatment was fed milk replacer with concentrate plus alfalfa.

To understand and integrate the correlations between the three high‐dimensional datasets (rumen epithelial and content microbiotas and the host transcriptome) in this study, multiple co‐inertia analysis (MCIA) (Meng *et al*., 2014) was performed. MCIA can simultaneously project several datasets into the same dimensional space according to a covariance optimization criterion. Figure 2A showed the projection of datasets of each animal onto the first two principle components (PCs) since the first and second axes of MCIA explained ∼80% of the variation across the multi‐omics datasets of samples according to the scree plot (Supporting Information Fig. S4). Across the same subject, three communities (rumen content community, epithelial community, host transcriptome) were connected by lines whose length represents the divergence (the shorter the line, the higher the level of concordance). Our results showed that three communities in MRO diets displayed the strongest concordance compared to solid diet treatments (Fig. 2A). We also observed that the similarity of rumen content and epithelial microbiota was greater than their similarities with the host transcriptome across diet treatments based on divergence. The pseudo‐eigenvalues associated with the first two PCs of each community are shown in Fig. 2B that summarized the contribution of each community to the total variance and the concordance between three communities. The transcriptome accounted for the highest variance on axis 1 and 2, while epithelial microbiota accounted for the second‐highest variance on axis 2 (pseudoeig 2). Moreover, the epithelial microbiota and host transcriptomic datasets were closed on the second axis, which indicates a strong association between the epithelium microbiota and its transcriptome (Fig. 2B). In short, solid diet could affect the rumen microbial community and transcriptome, and the epithelial microbiota may play more important roles for the host transcriptome.

To quantify the proportions of the host epithelial transcriptome and the rumen microbiota that were influenced by diet treatment, macro‐nutrient intake [crude protein (CP), neutral detergent fibres (NDF), and non‐fibrous carbohydrates (NFC)], rumen fermentation parameters [VFA, microbial crude protein (MCP)], epithelial morphology and impacts of the associated microbiota (pseudoeig 2 from MCIA was used due to variance of three communities), permutational multivariate analysis of variance (PERMANOVA) was performed. Solid diet was the most significant factor shaping rumen microbiota and the transcriptome. While NDF and NFC in solid diet were the major factors for the rumen content microbial community with 24% and 25% variation respectively, NDF was the only major factor that contributed to the epithelial microbiotas and the epithelium transcriptome, with approximately 24% and 32% variation (Table 1). Epithelial microbiota explained 13% of the variation in the transcriptome, while the rumen content microbiota could explain 2.65%. The effects of the content and epithelial microbiomes had similar effects with each other (8%–9% variation), which was consistent with their similarities in the MCIA analysis. Total VFAs were another significant factor for content and epithelial microbiotas, with about 7% variation explained. Then we performed an additional PERMANOVA model, excluding diet factors, for the detection of rumen fermentation effects (Supporting Information Table S4). Significant impacts regarding acetate and butyrate on the three datasets were observed, and similar variation accounted for VFAs and the associated microbiota. Briefly, intake of diet and its NDF nutrients were the biggest factor influencing rumen microbiota and the host transcriptome, and the epithelial microbiota had a stronger impact on rumen gene expression than content microbiota.

**Table 1:** PERMANOVA analysis of the factors affecting the rumen microbiota and host transcriptome (multivariate models).

Host transcriptome	*R* ^2^ (%)	*P*	Epithelial microbiota	*R* ^2^ (%)	*P*	Content microbiota	*R* ^2^ (%)	*P*
NDF intake	24.40	0.01	NDF intake	32.41	0.01	NDF intake	32.41	0.01
NFC intake	25.05	0.01	NFC intake	4.37	0.27	NFC intake	4.37	0.2
CP intake	4.91	0.17	CP intake	6.95	0.1	CP intake	6.95	0.04
EPC2	13.01	0.02	CPC2	8.62	0.01	EPC2	9.20	0.03
Total VFA	2.28	0.35	Total VFA	7.10	0.09	Total VFA	7.10	0.09
CPC2	1.97	0.42	Rumen weight	4.57	0.25	Rumen weight	5.12	0.17
Rumen weight	4.61	0.18	Papilla length	6.69	0.08	Papilla length	5.09	0.12
Papilla length	4.39	0.12	Papilla width	2.78	0.54	Papilla width	2.72	0.57
Papilla width	2.07	0.43						

Samples from rumen content microbiota, epithelial microbiota and host transcriptome were used to perform PERMANOVA analysis with two sequential orders listed in the table: intake of NDF, NFC, CP, PC2 of the microbiota, VFA, parameter of epithelial morphology (b).

Data were analysed using the ‘Vegan’ package in the R program. CPC2, content microbiota PC2 from multiple co‐inertia analysis; EPC2, epithelium microbiota PC2 from multiple co‐inertia analysis.

**Fig 3:**
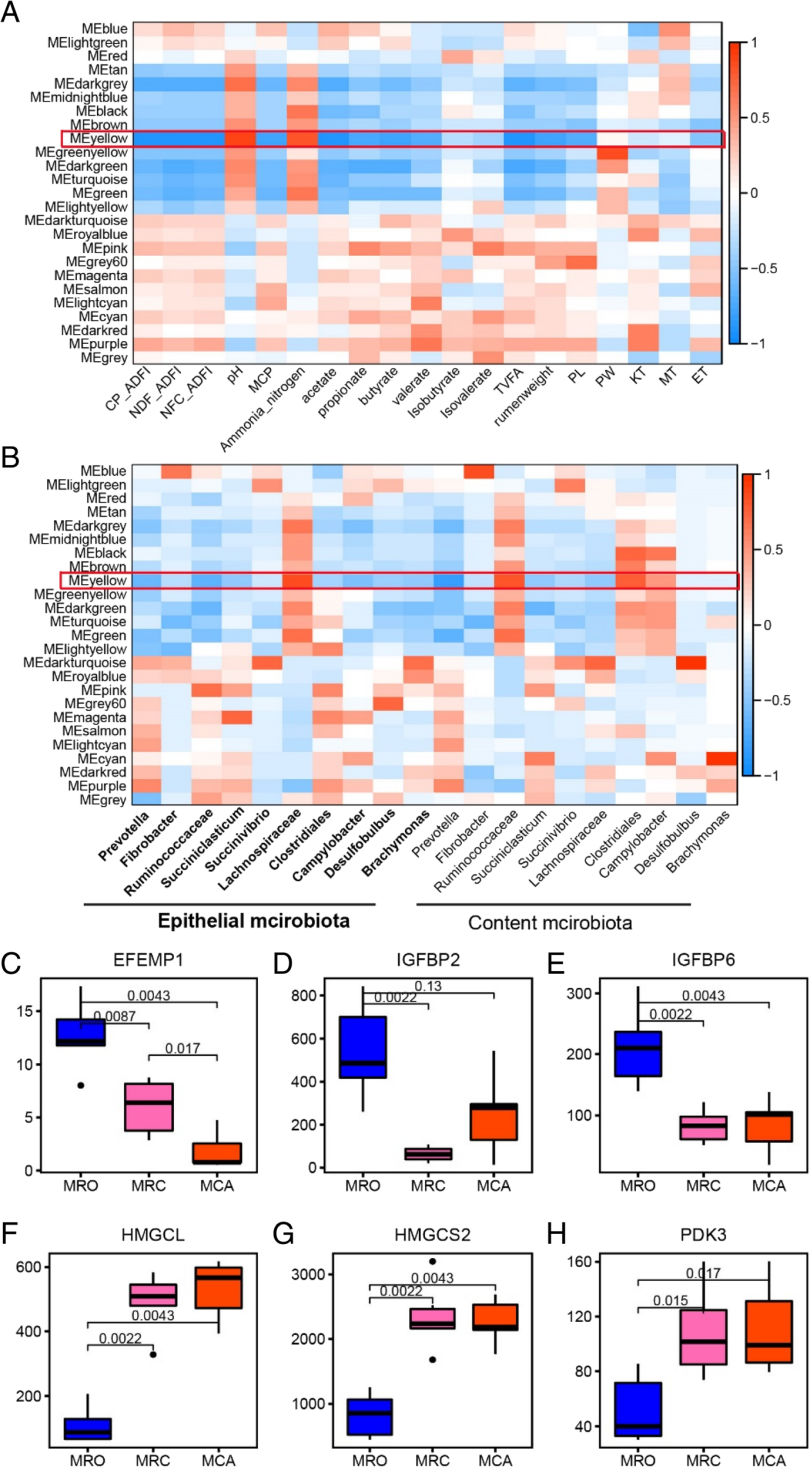
The correlation between WGNCA modules of the host transcriptome and goats' phenotypic traits as well as major genes' abundances in yellow modules. A. The correlation between the host transcriptome and animal phenotype. B. The correlation between the host transcriptome and the main genus in the epithelial and content communities. Blue colours represent negative correlations, whereas oranges colours represent positive correlations. Numerical values within a square represent Pearson correlation (upper value) and *P* value (lower value). C–H. The major gene abundances (FPKM) in the yellow module were associated with diet regimes. The MRO treatment was fed only milk replacer, the MRC treatment was fed milk replacer with concentrate and the MCA treatment was fed milk replacer with concentrate plus alfalfa. CP_ADFI: Crude protein average daily intake; NDF_ADFI: neutral detergent fibres average daily intake; NFC_ADFI: non‐fibrous carbohydrates average daily intake; MCP: rumen microbial proteins; VFA: volatile fatty acids; KT: keratin layer thickness; MT: muscle layers thickness; ET: epithelium thickness; EFEMP1: GF‐containing fibulin extracellular matrix protein 1; IGFBP2: insulin‐like growth factor binding protein 2; IGFBP6: insulin‐like growth factor‐binding protein 6; HMGCL: 3‐hydroxy‐3‐methylglutaryl‐CoA lyase; HMGCS2: 3‐hydroxy‐3‐methylglutaryl‐CoA synthase 2; PDK3: pyruvate dehydrogenase kinase 3.

To deeply understand the interactions between phenotypes and gene expression in epithelial tissue, a weighted gene co‐expression network analysis (WGCNA) was performed. Common host genes (17 989) expressed in all goats were clustered into 25 modules (defined as colours; Supporting Information Fig. S5 A). These genes displayed various associations with goat phenotypic traits. Yellow modules (1678 genes; 9.33% of total transcripts; Supporting Information Fig. S5B) yielded the strongest negative correlation with goats' phenotype and rumen microbiota (Fig. 3A and B). In the yellow module, host genes were related to ‘cell’, ‘cellular processes’, ‘binding’ and ‘biological regulation’ (Supporting Information Fig. S6, File S1). Moreover, major genes in the yellow module were differently expressed in diet groups. Genes, such as *EFEMP1*, *IGFBP2*, and *IGFBP6* (Fig. 3C–E) involved in rumen epithelial growth and solid diet stimuli (Supporting Information File S1), were down‐regulated in solid diet treatments compared to MRO. Several genes associated with carbohydrate and lipid metabolism pathways were up‐regulated, including *HMGCL*, *HMGCS2*, and *PDK3*, while other genes belonging to solute carrier (SLC) gene families (e.g. SLC6A14 and SLC6A15) were mostly down‐regulated in goat kids supplemented with solid diet (Fig. 3F–H; Supporting Information File S1). Other important down‐regulated genes were related to zinc finger proteins (*ZNF268*, *ZNF496*, *ZNF512B*, *ZNF521*, *ZNF532* and *ZNF618*; Supporting Information Fig. S7) that play an important role in early rumen papillae development and keratinization in goat kids (Supporting Information File S1). In short, through WGCNA, the major genes that strongly correlated with phenotypes and rumen bacteria were affected by solid diet.

**Fig 4:**
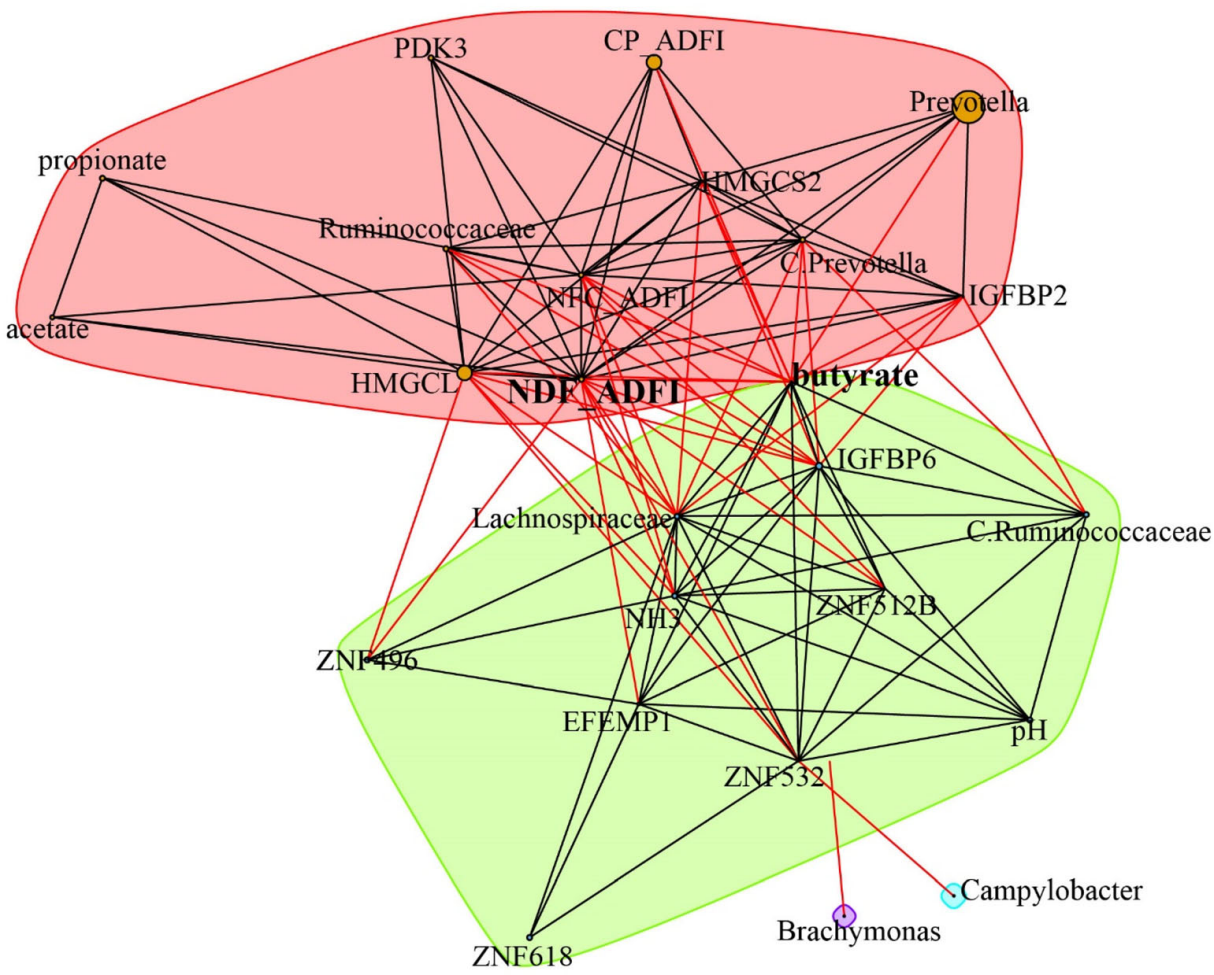
Network analysis of the interaction among host phenotypes, rumen microbiota and transcriptome. SparCC was used to calculate the relations between parameters. R packages were used to develop the network. CP_ADFI: Crude protein average daily intake; NDF_ADFI: neutral detergent fibres average daily intake; NFC_ADFI: non‐fibrous carbohydrates average daily intake. Bacteria in network plot were from the epithelial community, while bacterial names prefaced with ‘C.’ were from the rumen content community.

Finally, the complex interactions among major factors in diet, rumen fermentation parameters, rumen microbiota in both content and epithelial communities, and the host transcriptome were displayed in a network calculated using SparCC algorithm (Fig. 4). NDF intake and rumen butyrate served as hub features connecting the rumen microbiota and host genes. Five epithelial microbiotas, including *Prevotella*, *Ruminococcaceae*, *Lachnospiraceae*, *Brachymonas* and *Campylobacter*, were associated with the major genes in the yellow module. Moreover, two rumen content microbiotas (*Prevotella* and *Ruminococcaceae*) showed correlation with butyrate, epithelial microbiota and genes of rumen. Altogether, solid diet and its NDF could influence rumen content microbiota and butyrate concentration, which may have subsequently impacted epithelial microbiota, and then affected gene expression of rumen tissue, resulting in improved rumen development.

## Discussion

Greater rumen development could increase feed utilization and performance of young ruminants. This study, consistent with previous work, observed improved rumen development (e.g. longer papilla length and width, higher rumen weight) and distinct changes within the rumen microbiota and transcriptome by solid diet supplementation (Ma *et al*., 2017; Lin *et al*., 2019; Lv *et al*., 2019; Yang *et al*., 2019). The rumen rapidly undergoes significant morphological transformations and becomes a functional organ when supplemented with a solid diet, therefore generating large interests regarding diet utilization and serving as a unique model for the study of nutrient–microbiota–host interactions (Yang *et al*., 2019).

In this study, early solid diet supplementation in goat kids increased diversity and richness of the epithelial microbiota, and distinct clusters of community structure and membership were found. Several studies have evaluated the effects of diet on the epithelial microbiota in growing goats (Wetzels *et al*., 2015), adult sheep (Seddik *et al*., 2018) and cattle (Petri *et al*., 2013; Scharen *et al*., 2017; Wetzels *et al*., 2017; Petri *et al*., 2018). However, previous studies reported that high grain diets or acidosis diets decreased the epithelial microbiota alpha diversity compared to baseline diets in adult ruminants (Wetzels *et al*., 2017; Petri *et al*., 2018; Seddik *et al*., 2018). This is likely due to a greater portion of the epithelial microbiota in their adult control animals remaining attached to rumen papillae, as well as the reduction of rumen pH by carbohydrate nutrient intake that may dissolve some digesta adhesion. Our MRO goats consumed fluid diet and had an underdeveloped rumen with short papillae, resulting in a lower population of microbiota adhered to rumen epithelium. In the current study, significant differences of the diversity and richness between rumen content and epithelial microbiota were observed within all three diet feeding regimes, consistent with previous results in sheep, cattle and yak research (Malmuthuge *et al*., 2014; Liu *et al*., 2016; Scharen *et al*., 2017; Ren *et al*., 2020). *Prevotella* in both the content and epithelial communities in solid diet treatments maintained higher abundances when compared with our control goats, which indicated that diet could influence digesta microbiota and, subsequently, the epithelial microbiota. In particular, the epithelial bacterial community is dominated by *Campylobacter*, reported in our study and previous studies, and likely involved in rumen anatomical development and the metabolism of ammonia and volatile fatty acids (Jiao *et al*., 2015; Mao *et al*., 2015; Wetzels *et al*., 2017). The ventral sac of a solid diet group, especially MCA, may gather more solid digesta, resulting in different microenvironments (e.g., oxygen, pH) and microbial compositions between content and epithelial communities. Compared with the rumen content microbiota, epithelial bacteria might be more associated with nitrogen utilization, oxygen scavenging, epithelial cell proliferation and VFA absorption through the rumen wall by modulating the expression of genes responsible for the absorptive processes in the rumen epithelium rather than in carbohydrate fermentation (Chen *et al*., 2012; Mao *et al*., 2015; Wetzels *et al*., 2017; Ren *et al*., 2020). Therefore, microbiota in rumen content and epithelial communities were affected by solid diet and had significant differences.

Investigation of microbiota–host interactions could allow us to better understand microbial functions. Our results confirmed that the epithelial microbiota potentially affected by a solid diet play more important roles in the host transcriptome and rumen development than rumen content microbiota. This might be possible since the epithelial microbiota directly contacts rumen papillae. Hence, the association between rumen content and the epithelial microbiota should be investigated in future research. The associations between goats' phenotypes and microbiota–host interaction were also deeply investigated. We confirmed that a solid diet, especially NDF and NFC, and its associated products (butyrate and acetate) are the most significant factors influencing rumen development. Previous studies in lambs confirmed that starter influenced host regulated, growth‐associated genes and papillae development by influencing the rumen content microbiota and its products such as butyrate and acetate (Lane and Jesse, 1997; Lin *et al*., 2019). Another study reported that alfalfa promotes rumen development through the regulation of gene expression related to metabolic processes and calcium transduction in lambs compared to only starter supplementation (Yang *et al*., 2019). Therefore, diet, especially NDF, could significantly influence the rumen content microbiota (Lv *et al*., 2019), and subsequently the epithelial microbiota and epithelium transcriptome.

It is suggested that solid diet is the main driver for rumen development. The identified host genes in the yellow module of WGCNA that were highly correlated with phenotypes provided a common ground to identify diet‐microbiota–host interactions and their potential regulatory mechanisms in the developing rumen. The numbers of host genes differently expressed in the yellow module were similar with previous studies (Connor *et al*., 2013; Malmuthuge *et al*., 2019). Moreover, we found *HMGCL* and *HMGCS2* were up‐regulated with solid diet supplementation. Conversion from HMG‐CoA to acetoacetate in ketogenesis by *HMGCL* is a hallmark of epithelium metabolic development (Wang *et al*., 2016). *HMGCS2*, which encodes a mitochondrial enzyme that catalyses the first reaction of ketogenesis, was down‐regulated with increasing NDF to starch ratio in dairy cows (Ma *et al*., 2017). The down‐regulated solute carrier genes (SLC families) are associated with VFA diffusion and other nutrients (Kong *et al*., 2016), which provided reasoning for more digesta to be attached on the epithelial walls. Most genes related to zinc finger proteins were also down‐regulated. *ZNFs* are host transcriptional factors that regulate a wide array of functions, including ‘recognition of DNA,’ ‘packaging of RNA,’ ‘activation of transcription,’ ‘protein folding and assembly,’ and ‘regulation of apoptosis’ (Laity *et al*., 2001). The *IGFBP2* genes that are upregulated in cancer tissue (Li *et al*., 2020) were found to be lower in solid diet groups, which indicated our control rumen development did not reach maturity when only consumed liquid diet. Therefore, a solid diet could potentially manipulate rumen microbiota, and the fermentation products of a solid diet and associated microbiota could regulate these genes to improve rumen tissue development. Early solid diet supplementation can also improve rumen development by physical stimulation (Vi *et al*., 2004; Diao *et al*., 2019). Recently, a study investigated the effects of physical stimuli from forage sources and particle size on rumen gene expression in dairy calves and reported the down‐regulated gene *EFEMP1* coding an epidermal growth factor family, a peptide motivating epithelial proliferation and differentiation when provided to the luminal side of the rumen, sustaining mucosal integrity and accelerating the healing of epithelial injuries (Wang *et al*., 2017). The *EFEMP1* gene in our yellow module was also down‐regulated by solid diet supplementation, indicating its association with a solid diet stimulus.

In the present study, the complex pathway from solid diet to rumen fermentation and microbiota to epithelial genes' expression was summarized. The NDF in solid diet was the major driver for the rumen environment, and the subsequent significant changes of butyrate and bacteria in the rumen content and epithelial communities highly impacted on host transcriptome. Although the composition of the epithelial microbial community and its importance on rumen gene expression were summarized in comparison with the rumen content community, the movement of digest or microbiota and volatile fatty acids in the rumen is also necessary to investigate, which would help facilitate our understanding of the rumen microbiome and their functions. Moreover, future studies should focus on species levels of rumen microbiota and its interaction with the host using metabolomics and metagenomics, likely of considerable importance to completely understand the roles of the rumen microbiota on early rumen development.

## Conclusions

This study investigated the microbial composition of rumen epithelial communities and the transcriptome from three diet regimes. Compared with content, microbiota attached on epithelium were distinct although shared dominant bacteria were found. Solid diet and its NDF contributed to manipulation of the rumen microbiota. The epithelial microbiota played a more important role to the host transcriptome and rumen development compared to rumen content microbiota. Future researchers should focus more attention on microbiota attached on epithelium.

## Experimental procedures

### Animal treatments and sampling

The experimental protocols in this study were approved by the Chinese Academy of Agricultural Sciences Animal Ethics Committee (Protocol Number: AEC‐CAAS‐FRI‐CAAS20180305; Approval date: 9 March 2018). A total of 72 Yangtze River Delta White Goat kids (20 days old) were selected from a commercial farm in the Jiangsu province, China. The goats were weaned and randomly assigned to three diet treatments: milk replacer only (MRO), milk replacer supplemented concentrate (MRC) and milk replacer supplemented concentrate plus alfalfa pellets (MCA). Each group contained six replicates and four kids per pen were used as replicates. During the animal trial, all goat kids had *ad libitum* access to water, the MRC and MCA kids were freely given access to concentrate, and the MCA goats were provided the additional free choice of alfalfa pellets.

At 60 days of age, six goat kids per treatment were slaughtered for rumen sample collection. Samples of rumen tissue at the bottom of the ventral sac were collected within 30 min post‐euthanasia. Tissue (~ 10 cm^2^) was washed using bacteria‐free PBS (pH = 7) to rinse the rumen content or fluid filling the gap between papillae. Residues attached tightly in the epithelium were abraded out for measurement of the epithelial microbiota. Concurrently, tissue sections (~ 4 cm^2^) in the ventral sac were fixed in a solution of 10% formalin for epithelial morphology detection. Samples for the remaining tissue and the epithelium‐associated microbiota were snap‐frozen in liquid nitrogen and stored at −80°C for host transcriptome and 16S rRNA sequencing, respectively.

### Measurement of rumen epithelial morphology

Rumen tissue sections were stored in 70% ethanol until further processing after 24 h of fixing in formalin. All samples were embedded in paraffin blocks and were stained with Yihong‐haematoxylin (H.E.) at the Chinese Agriculture University (Beijing, China). The length and width of the rumen papillae and stratum corneum thickness were measured using the Axiovision software (Zeiss, Oberkochen, Germany) Image‐pro express image analysis processing system.

### Analysis of rumen epithelial microbiota affected by diet treatments

Total epithelial microbial DNA was extracted, and the V3‐V4 region of the bacterial 16S ribosomal RNA genes was amplified using indexes and adaptor‐linked universal primers (431F and 806R). Amplicon libraries were mixed using all qualified products and sequenced with an Illumina HiSeq PE250 platform at the Realbio Technology Genomics Institute (Shanghai, China). More details related to the sequencing process can be found in our previous study (Lv *et al*., 2019).

Rumen content microbiota data of the same goat kids were obtained from our previously published work Lv *et al*. (Lv *et al*., 2019). Raw sequences of the rumen content and epithelial microbiota were analysed using the program mothur (v1.39.1) (Kozich *et al*., 2013). Contigs were combined from two sets of reads from all samples. The sequences were aligned using the SILVA reference database (full‐length sequences and taxonomy references release 132; http://www.arb‐silva.de/documentation/release‐132/) (Pruesse *et al*., 2007). Chimeras in filtered sequences were removed using the VSEARCH algorithm. High‐quality sequences were classified into operational taxonomic units (OTUs) at the 97% similarity level using the Ribosomal Database Project (RDP) database (Cole *et al*., 2009). Alpha diversities (Shannon index and Observed OTUs) and beta diversities (Bray–Curtis and Jaccard distance) were calculated. The boxplots of alpha diversity and the PCoA plot of beta diversity were visualized using the ‘ggplot2’ package in R (v3.6.0). The ANOSIM test was used to detect the statistical significance of beta diversity. The microbial sequencing data in the current study are available from the NCBI SRA database with BioProject ID PRJNA594390.

### Transcriptome profile of rumen epithelium tissue affected by diet treatment

Total RNA of rumen tissue samples was extracted using the TRIzol reagent according to the manufacturer's protocol (Invitrogen, CA, USA). The integrity and concentration of RNA were measured using an Agilent 2100 Bioanalyzer to verify the integrity number to be greater than 7, and the library was prepared for RNA‐seq using the TrueSeq RNA Sample Preparation Kit v2 (Illumina, CA, USA) to enrich poly‐A tailed host mRNA with oligodT beads. RNA libraries were sequenced at the Beijing Genomics Institution (Shenzhen, China) using the HiSeq 2000 system (Illumina) to obtain 100‐bp paired‐end reads according to the manufacturer's instructions.

Raw reads were filtered to obtain clean reads using the trimmomatic module in SOAPnuke (v1.4.0) for the removal of adaptor contamination and low‐quality reads (more than 20% of bases with a quality score smaller than 10, or having more than 5% ambiguous sequences labelled as ‘N’). Then, clean RNA reads were mapped and assembled to reference genomes (AnimalTFDB v2.0) using HISAT (v2.1.0) (Kim *et al*., 2015). The detection of transcript expression levels was based on the number of fragments per kilobase of exon per million fragments mapped (FPKM). Differentially expressed genes (DEGs) were detected based on methods reported by Wang *et al*. (2010) and the false discovery rate (FDR) was calculated based on methods of Benjamini and Hochberg's multiple testing correction (Benjamini and Hochberg, 1995). The significantly DEGs were confirmed at a fold change ≥ 2 and a FDR < 0.001. Using this method, the DEGs were displayed through a pairwise comparison analysis (MRO‐vs‐MRC, MRO‐vs‐MCA and MRC‐vs‐MCA). After expression pattern clustering, the DEGs from pairwise comparisons were subjected to functional annotation, including GO functional annotation and Kyoto Encyclopedia of Genes and Genomes (KEGG) pathway annotation. The GO terms and KEGG pathway enrichment were performed using The Database for Annotation, Visualization and Integrated Discovery (DAVID v 6.8, http://david.abcc.ncifcrf.gov) (Huang *et al*., 2009). The RNA‐seq data are available in the NCBI SRA database (BioProject ID PRJNA594390).

### Bioinformatics for high‐dimensional datasets of rumen microbiota and transcriptome associated with diet treatments

The general association between the rumen content, epithelial microbiota and host tissue transcriptome was studied using MCIA in R ‘omicade4’ packages (v 1.26.0). All pipelines used the custom script (Meng *et al*., 2014). PERMANOVA was performed to disclose the factors shaping the rumen microbiota (content and epithelial) and host transcriptome. We used the ‘adonis’ function in the ‘vegan’ package of R including different independent variables (e.g., diet, pseudoeig 2 of rumen microbiota from MCIA, VFA and epithelial morphology parameters) with default settings (Bray–Curtis distance and 99 permutations).

The interactions among host gene expression, the rumen content microbiota and epithelial microbial communities were explored through Weighted gene co‐expression network analysis (WGCNA) (Langfelder and Horvath, 2008). All expressed protein‐coding genes (17 989 FPKM) in rumen tissue samples collected from all goats were used in the WGCNA analysis, respectively (R package v3.6.0). First, a weighted network was built based on the co‐expression patterns among genes/OTUs using the ‘pickSoftThreshold’ function in the ‘WGCNA’ package. Then, the ‘blockwiseModules’ function was used to build the network and detect modules using a hierarchical clustering approach. The parameters for module detection were maxBlockSize of 18 000, minModuleSize of 30, reassignThreshold of 0 and verbose of 3. This approach generated 25 transcriptome modules (defined as colours). We measured the link between the host transcriptome and the goat phenotypic traits (macro‐nutrient intake, the concentration of acetate, propionate, butyrate, valerate, isobutyrate, isovalerate, total VFA, and papillae length and width) and rumen microbiota. The Pearson correlation coefficients between the eigengenes for each gene/OTUs module and goats' phenotypic traits were calculated. The boxplot of the main genes in the yellow module that were highly correlated with phenotypes and rumen microbiota were made in R.

The SparCC algorithm that is able to estimate the correlations from a compositional network was used for network analysis. The network was demonstrated using the ‘igraph’ package in R with edges connecting nodes with a correlation co‐efficiency over 0.5 or less than − 0.5.

## Author contributions

Q.D. and N.Z. developed the experimental design and managed grant. J.C. and N.Z. contributed to all data analysis, results interpretation and manuscript writing. X.L. and K.C. contributed to animal trials, sample collection and next generation sequencing. J.C., H.U. and N.Z. contributed to drafting and proofing. W.H. and Y.Z. helped in sample collection. All authors have reviewed this manuscript and agree to its final version.

## Supporting information


**Table S1.** Effect of early supplementary solid diet on growth performance of goat kids.
**Table S2.** Effects of early feeding on the development of rumen morphology in goat kids.
**Table S3** ANOSIM results based on Bray‐Curtis distance.
**Table S4.** PERMANOVA analysis of the factors affecting the rumen microbiome and host transcriptome (multivariate models).
**Fig. S1.** Solid diet intervention influences the rumen papilla development and epithelium gene transcriptome.
**Figure S2.** Venn plot of total differently expressed genes (DEG) in pair‐wise comparisons.
**Figure S3.** GO enrichment analysis of differently expressed genes (DEG) in three pair‐wise comparisons.
Figure S4.
**Figure S5.** The gene module classified by WGCNA.
**Figure S6.** WGCNA enriched functions of yellow modules.
**Figure S7.** The major genes abundances (FPKM) in yellow module associated with diet regimes.

**File S1.** genes in yellow WGCNA module and their functions

## Data Availability

The sequencing data in the current study are available in the NCBI SRA database with the BioProject ID PRJNA594390.
