# Supplementary material for: Solid diet manipulates rumen epithelial microbiota and its interactions with host transcriptomic in young ruminants
Source: Environ Microbiol. 2021 Sep 20;23(11):6557–68. doi: 10.1111/1462-2920.15757 (PMC9292864; doi:10.1111/1462-2920.15757)

**Solid Diet Manipulates Rumen Epithelial Microbiota and Its Interactions with Host Transcriptomic in Young Ruminants**

Jianmin Chai^12^, Xiaokang Lv^1^, Qiyu Diao^1^, Hunter Usdrowski^2^, Yimin Zhuang^1^, Wenqin Huang^1^, Kai Cui^1^, Naifeng Zhang^1^**^#^**

^1^Feed Research Institute of Chinese Academy of Agricultural Sciences, Key Laboratory of Feed Biotechnology of the Ministry of Agriculture and Rural Affairs, Beijing 100081, China

^2^Department of Animal Science, Division of Agriculture, University of Arkansas, Fayetteville, AR, 72701, USA

**^#^Correspondence:**

Professor Naifeng Zhang, Email zhangnaifeng@caas.cn

Postal address: No.12 Zhongguancun South St., Haidian District, Beijing, P.R.China 100081

Phone #: +86(10) 6216 6878

Fax #: +86(10) 6216 9105

Table S1 Effect of early supplementary solid diet on growth performance of goat kids

|  | Treatments | | | SEM |  |
| --- | --- | --- | --- | --- | --- |
| Items | MRO | MRC | MCA |  | *P*-value |
| Milk replacer intake/(g•d^-1^) | 130.62 | 107.91 | 119.90 | 4.95 | 0.1696 |
| Concentrate intake/(g•d^-1^) | --- | 188.18 | 189.56 | 4.82 | 0.8956 |
| Alfalfa pellets intake/(g•d^-1^) | --- | --- | 34.34 | --- | --- |
| Dry matter intake/(g•d^-1^) | 122.47^c^ | 271.50^b^ | 309.97^a^ | 16.12 | <0.0001 |
| Protein intake/(g•d^-1^) | 31.33^c^ | 58.52^b^ | 66.37^a^ | 4.07 | <0.0001 |
| NDF-Neutral detergent fiber intake/(g•d^-1^) | --- | 49.25^b^ | 66.08^a^ | 3.13 | 0.0004 |
| NFC-Non-fibrous carbohydrate intake/(g•d^-1^) | 63.82^c^ | 118.16^b^ | 131.15^a^ | 7.94 | <0.0001 |

MRO=milk replacer, MRC= milk replacer + concentrate, MCA= milk replacer + concentrate + alfalfa; SEM = Standard error of the means.

In the same row, values with different small letter superscripts mean significant difference (*P*＜0.05).

Table S2 Effects of early feeding on the development of rumen morphology in goat kids

| Items | MRO | MRC | MCA | SEM | P value |
| --- | --- | --- | --- | --- | --- |
| Slaughter BW, kg | 7.01^b^ | 10.47^a^ | 10.23^a^ | 0.41 | <0.000 1 |
| Average daily gain/g | 70.28^b^ | 123.93^a^ | 126.25^a^ | 5.48 | <0.0001 |
| Rumen weight/g | 47.42^c^ | 126.70^b^ | 175.67^a^ | 14.94 | <0.001 |
| Papilla length /μm | 786.6^b^ | 1191.6^a^ | 1389.6^a^ | 80.44 | 0.0008 |
| Papilla width /μm | 169.81^b^ | 206.52^ab^ | 237.13^a^ | 9.85 | 0.0096 |
| Keratin layer thickness /μm | 33.68 | 49.98 | 36.75 | 3.38 | 0.1258 |
| Muscle layers thickness /μm | 698.60 | 628.40 | 861.30 | 47.28 | 0.1171 |
| Epithelium thickness /μm | 43.13^b^ | 56.07^a^ | 47.72^ab^ | 2.19 | 0.0400 |

Superscripts a, b and c denote differences between treatments (P < 0.05);

MRO=milk replacer; MRC= milk replacer + concentrate; MCA= milk replacer + concentrate + alfalfa

Table S3 ANOSIM results based on Bray-Curtis distance

| Epimural microbiome | R | P value | Biogeography | R | P value |
| --- | --- | --- | --- | --- | --- |
| MCAE-MRCE | 0.07 | 0.228 | MCAC-MCAE | 0.73 | 0.003 |
| MCAE-MROE | 0.92 | <0.001 | MRCC-MRCE | 0.51 | 0.005 |
| MRCE-MROE | 0.86 | 0.003 | MROC-MROE | 0.99 | 0.001* |

ANOSIM: Analysis of similarities. MROC, MRCC and MCAC means content microbiome in animals received MRO, MRC and MCA diets, while MROE, MRCE and MCAE represent the epimural microbiome from three diets. MRO=milk replacer; MRC= milk replacer + concentrate; MCA= milk replacer + concentrate + alfalfa.

Table S4 PERMANOVA analysis of the factors affecting the rumen microbiome and host transcriptome (multivariate models)

| Host Transcriptome | R2 | P | Epimural microbiome | R2 | P | Content microbiome | R2 | P |
| --- | --- | --- | --- | --- | --- | --- | --- | --- |
| EPC2 | 18.20% | 0.02 | CPC2 | 26.72% | 0.01 | EPC2 | 26.12% | 0.01 |
| Butyrate | 18.30% | 0.01 | Butyrate | 14.13% | 0.02 | Butyrate | 15.04% | 0.02 |
| Acetate | 17.06% | 0.02 | Acetate | 8.85% | 0.03 | Acetate | 8.76% | 0.05 |
| Propionate | 3.70% | 0.27 | Propionate | 6.17% | 0.09 | Propionate | 6.21% | 0.17 |
| Rumen weight | 1.94% | 0.46 | Rumen weight | 3.54% | 0.45 | Rumen weight | 3.68% | 0.4 |
| Papilla length | 12.22% | 0.02 | Papilla length | 5.63% | 0.19 | Papilla length | 5.29% | 0.18 |
| Papilla width | 1.29% | 0.79 | Papilla width | 1.92% | 0.87 | Papilla width | 1.99% | 0.89 |
| CPC2 | 2.67% | 0.39 |  |  |  |  |  |  |

PERMANOVA: Permutational multivariate analysis of variance. EPC2: Epithelium microbiome PC2 from Multiple Co-inertia Analysis; CPC2: Content microbiome PC2 from Multiple Co-inertia Analysis.

Fig. S1 Solid diet intervention influences the rumen papilla development and epithelium gene transcriptome.


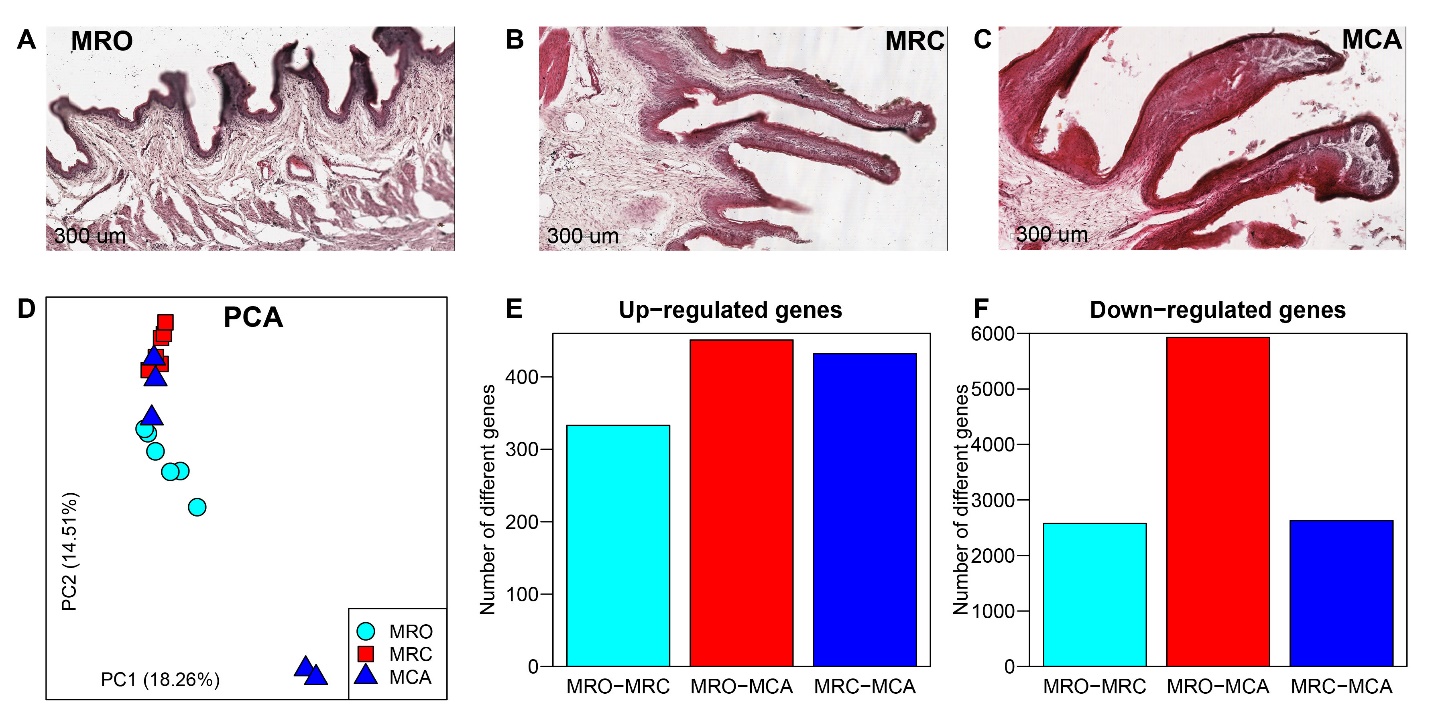


Rumen papillae development in goats that received MRO (A), MRC (B) and MCA (C) diets. Images are obtained from a light micrograph of rumen tissue at a magnification of × 10 objective lens (bar = 300 μm). D: PCA plot of the rumen epithelial transcriptomes from goats received three diet regimes. E: The number of up regulated genes in pair wise comparison (333 in MRO-vs-MRC, 451 in MRO-vs-MCA and 432 in MRC-vs-MCA). F: The number of down regulated genes in pair wise comparison (2575 in MRO-vs-MRC, 5926 in MRO-vs-MCA and 2630 in MRC-vs-MCA).

MRO=milk replacer; MRC= milk replacer + concentrate; MCA= milk replacer + concentrate + alfalfa.

Figure S2 Venn plot of total differently expressed genes (DEG) in pair-wise comparisons


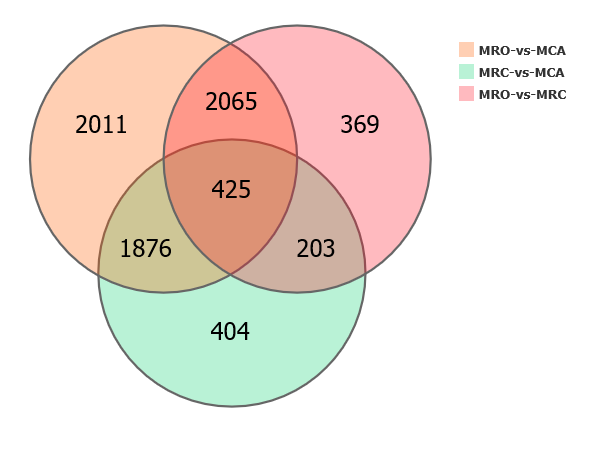


MRO=milk replacer; MRC= milk replacer + concentrate; MCA= milk replacer + concentrate + alfalfa.

Figure S3 GO enrichment analysis of differently expressed genes (DEG) in three pair-wise comparisons


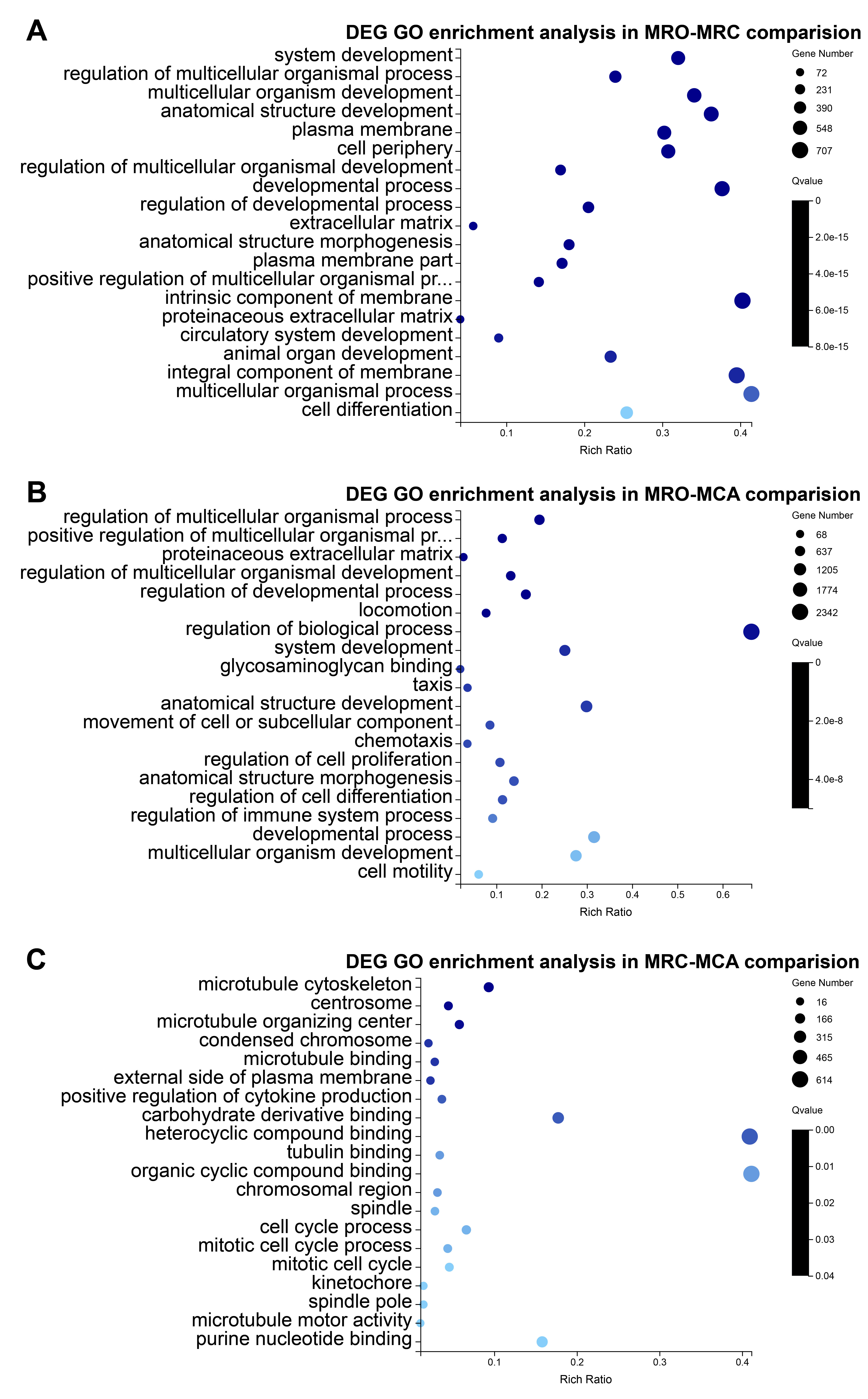


Differential Gene Ontology enrichment bubble map in comparison of MRO-vs-MRC (A), MRO-vs-MCA (B) and MRC-vs-MCA (C). The x axis displays the rich ration, and y axis shows the GO term. The size of the bubble represents the numbers of DEG in the specific GO term. The color density represents the Qvalue of the rich ratio, and the deeper blue means a smaller Qvalue.

Figure S4


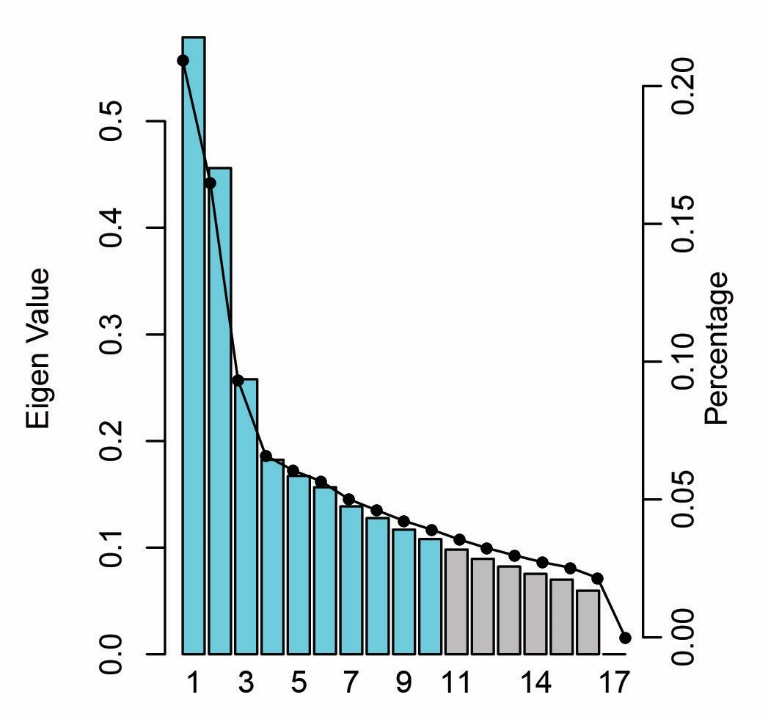


Pseudo-eigenvalues of three communities in this study, including rumen content and epithelial microbiota and transcriptome. Each pseudo-eigenvalue is associated with a principal component (linearly uncorrelated variables defined by MCIA) indicating the variance explained by each PC. Barplot shows the pseudo-eigenvalues (left axis) and black line corresponds to the percentage of variance of each PC, calculated as the eigenvalue divided by the sum of all eigenvalues.

Figure S5 The gene module classified by WGCNA


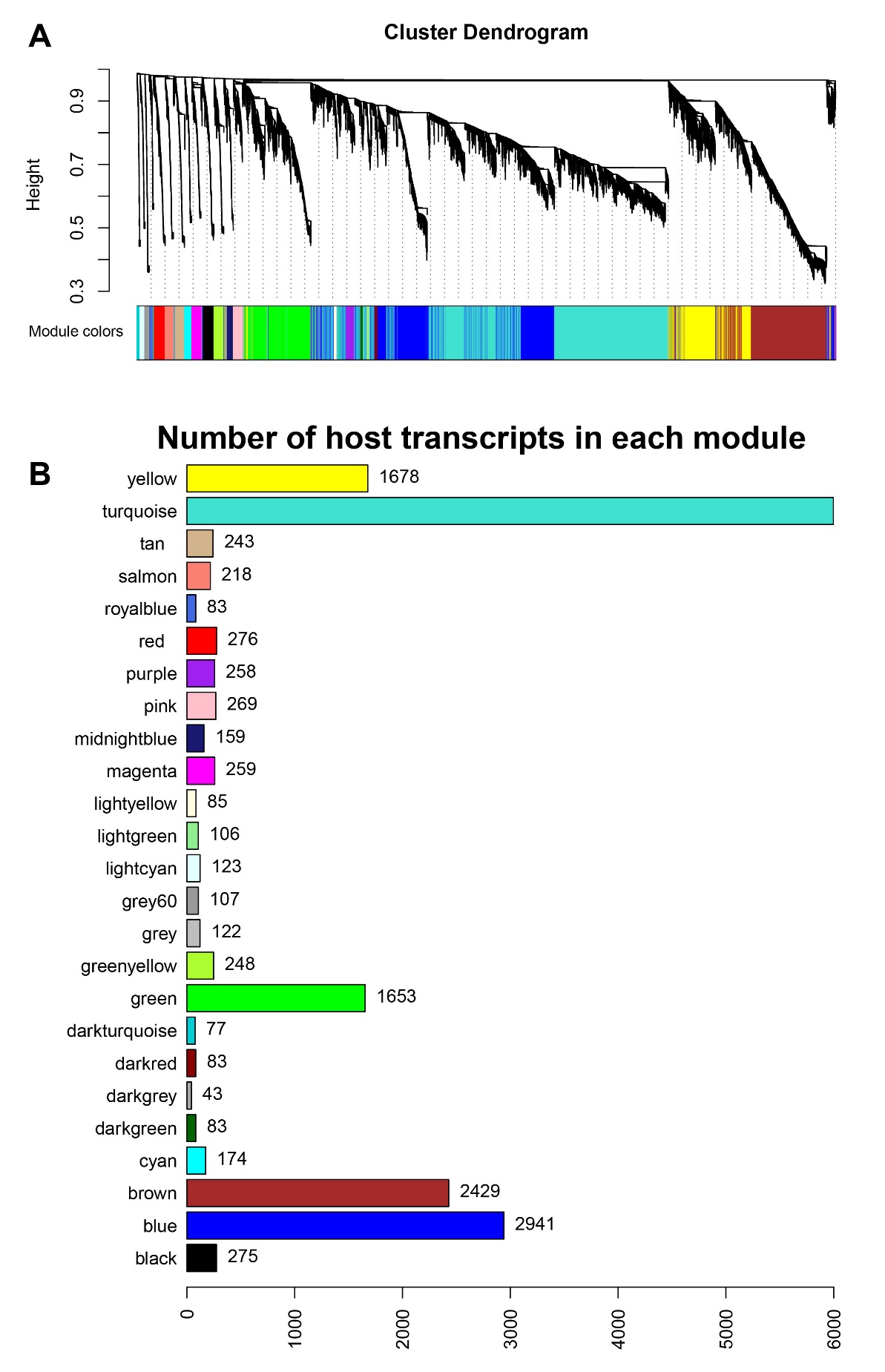


A: Dendrogram from gene co-expression network analysis of samples among the 3 diet groups. Modules of co-expressed genes were assigned a color and number (M1 to M25

B: gene numbers in each module

WGCNA: weighted gene co-expression network analysis.

Figure S6 WGCNA enriched functions of yellow modules


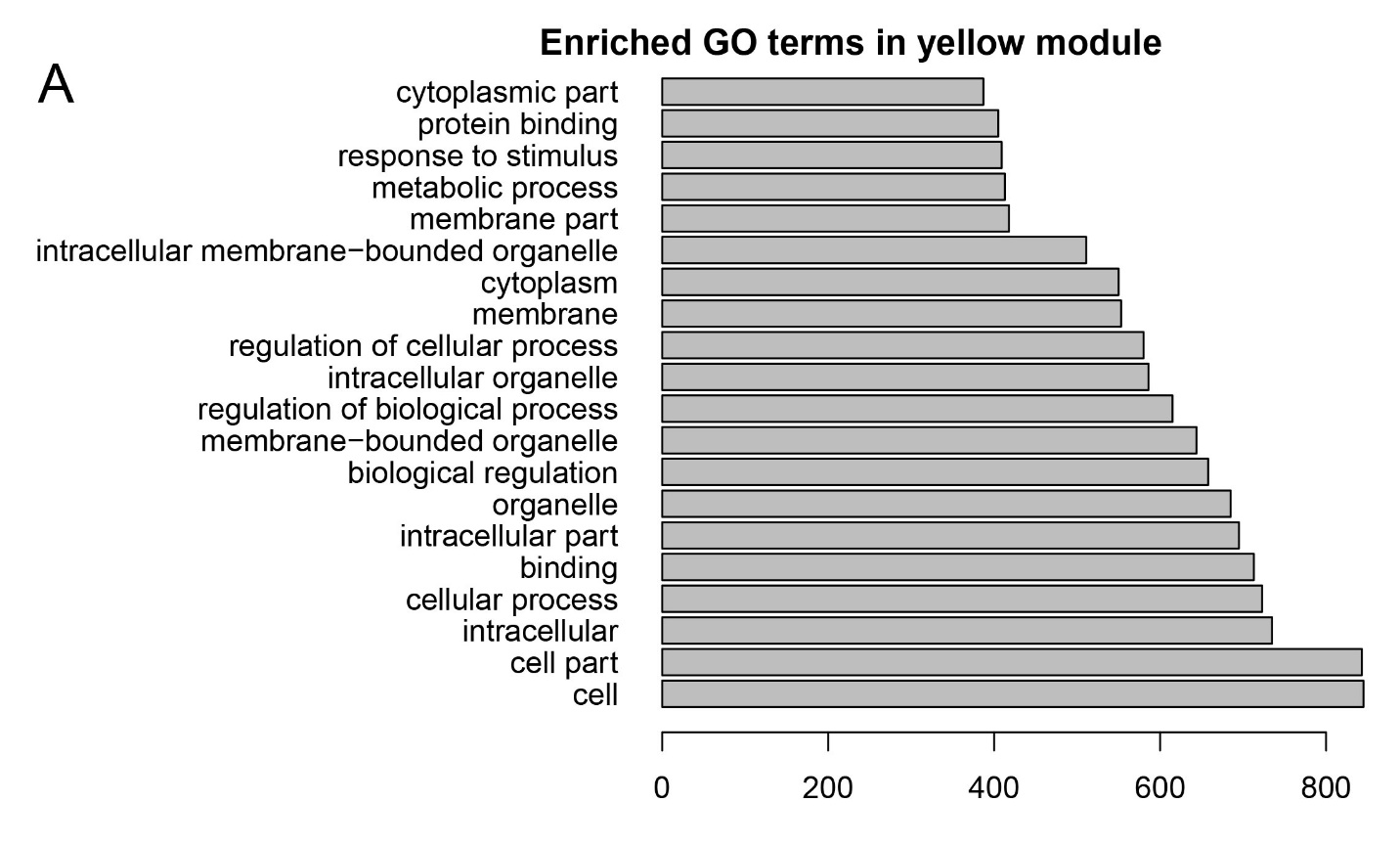


WGCNA: weighted gene co-expression network analysis.

Figure S7 The major genes abundances (FPKM) in yellow module associated with diet regimes


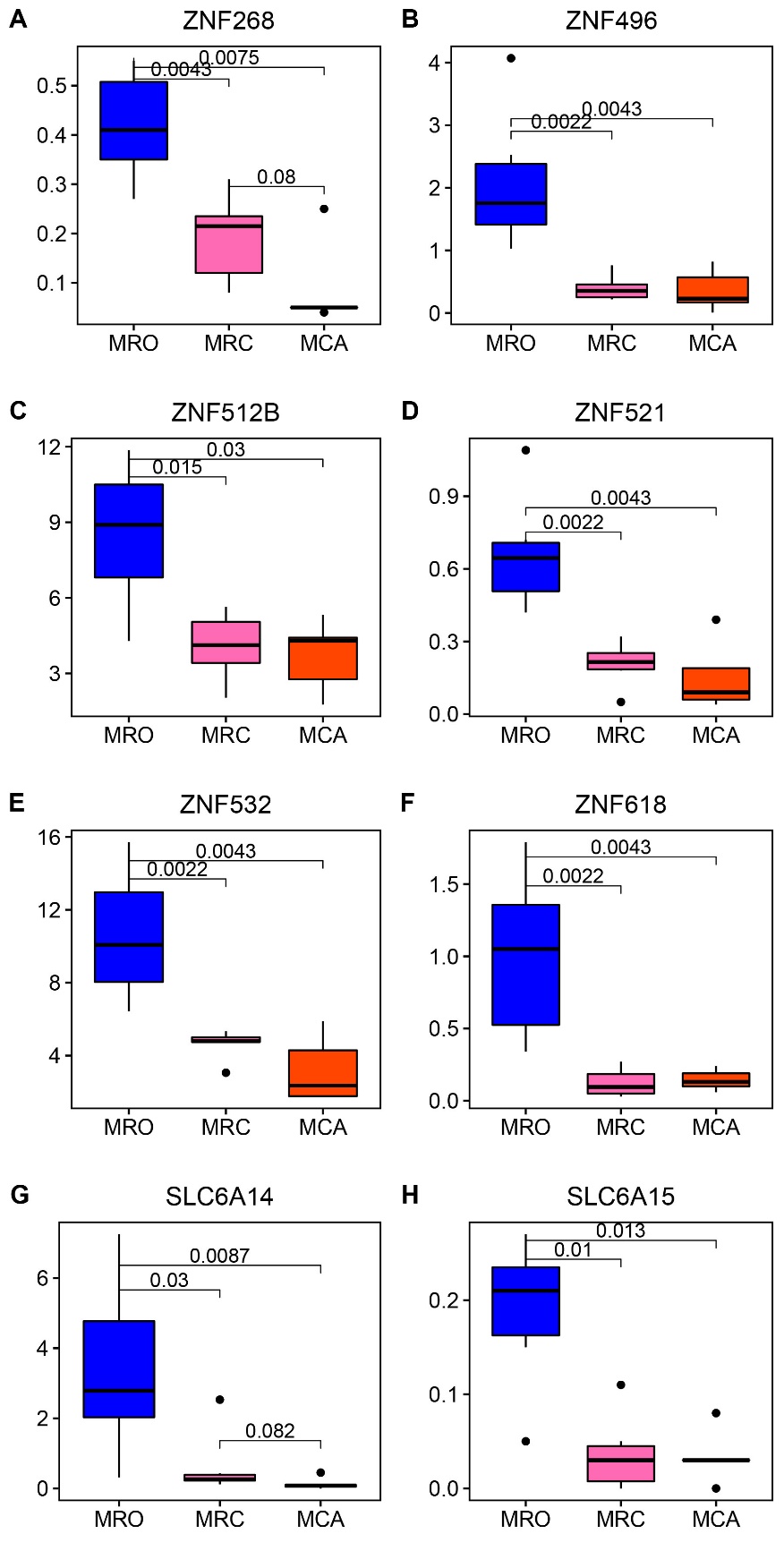

Supplement: Supplementary file 1 — Table S1. Effect of early supplementary solid diet on growth performance of goat kids. Table S2. Effects of early feeding on the development of rumen morphology in goat kids. Table S3 ANOSIM results based on Bray‐Curtis distance. Table S4. PERMANOVA analysis of the factors affecting the rumen microbiome and host transcriptome (multivariate models). Fig. S1. Solid diet intervention influences the rumen papilla development and epithelium gene transcriptome. Figure S2. Venn plot of total differently expressed genes (DEG) in pair‐wise comparisons. Figure S3. GO enrichment analysis of differently expressed genes (DEG) in three pair‐wise comparisons. Figure S4. Figure S5. The gene module classified by WGCNA. Figure S6. WGCNA enriched functions of yellow modules. Figure S7. The major genes abundances (FPKM) in yellow module associated with diet regimes. [file EMI-23-6557-s002.docx]
